# Assessment of Potential Use of a Composite Based on Polyester Textile Waste as Packing Elements of a Trickle Bed Bioreactor

**DOI:** 10.3390/ma17092028

**Published:** 2024-04-26

**Authors:** Martyna Gloc, Katarzyna Paździor, Marcin Kudzin, Zdzisława Mrozińska, Iwona Kucińska-Król, Renata Żyłła

**Affiliations:** 1 Lukasiewicz Research Network-Lodz Institute of Technology, 19/27 Marii Sklodowskiej-Curie Street, 90-570 Lodz, Polandzdzislawa.mrozinska@lit.lukasiewicz.gov.pl (Z.M.);; 2 Department of Bioprocess Engineering, Faculty of Process and Environmental Engineering, Lodz Univeristy of Technology, 213 Wolczanska Street, 90-924 Lodz, Poland

**Keywords:** biological treatment, circuit economy, packing media, polyester waste, textile waste, wastewater, waste management

## Abstract

Biological wastewater treatment using trickle bed reactors is a commonly known and used solution. One of the key elements of the proper operation of the trickle bed bioreactor is the appropriate selection of biofilm support elements. The respective properties of the bioreactor packing media used can influence, among other things, the efficiency of the treatment process. In this study, the possibility of polyester waste material usage for the preparation of the biofilm support elements was tested. The following properties were checked: adsorption capacity, swelling, surface morphology, microbicidal properties, as well as the possibility of their use in biological wastewater treatment. The tested elements did not adsorb copper nor showed microbicidal properties for bacterial strains *Escherichia coli* and *Staphylococcus aureus* as well as fungal strains *Aspergillus niger* and *Chaetomium globosum*. The hydrophilic and rough nature of the element surface was found to provide a friendly support for biofilm formation. The durability of the elements before and after their application in the biological treatment process was confirmed by performing tests such as compressive strength, FTIR analysis, hardness analysis and specific surface area measurement. The research confirmed the applicability of the packing elements based on polyester textile waste to the treatment of textile wastewater. The treatment efficiency of the model wastewater stream was above 90%, while in the case of a stream containing 60% actual industrial wastewater it was above 80%. The proposed solution enables the simultaneous management of textile waste and wastewater treatment, which is consistent with the principles of a circular economy. The selected waste raw material is a cheap and easily available material, and the use of the developed packing elements will reduce the amount of polyester materials ending up in landfills.

## 1. Introduction

Human operations are influencing the constantly degraded state of the environment [1]. Increasing amounts of waste in landfills, scarcity of drinking water and generation of large amounts of industrial wastewater are just a few of the environmental threats facing mankind.

For years, there has been an increasing trend of treating and reusing wastewater and recycling more and more solid waste. This means that both wastewater and other wastes are no longer seen merely as a material to be utilised but as a raw material that is the starting point for the creation of a new product [2,3]. Currently, there are many wastewater treatment methods available on the market, including adsorption [4], biological processes [5], membrane filtration [6], coagulation and flotation [7], ion exchange [8], ozonation [9], photocatalysis [10] or electrochemical methods. However, there is still an urgent need to develop new technological solutions to treat produced wastewater streams in an economically and environmentally attractive manner [11]. Methods can be appropriately matched to the composition of industrial wastewater, which is closely related to the type of plant activity from which it originates [12].

The textile industry significantly contributes to environmental pollution. Textile production requires enormous consumption of water, chemicals and energy [13,14,15,16]. According to data for 2020, the textile industry was responsible for approximately 20% of global water pollution [14,17]. Another significant problem in the treatment of generated wastewater streams is the inability to determine their universal composition, which is strictly dependent on the substrates and technological processes used during textile processing and finishing [18,19]. Depending on the technological process or one of its stages, streams differ in concentration, type of pollutants and the amount of wastewater produced [20].

Wastewater from the textile industry is characterised by high loads of organic and inorganic compounds, including: dyes, surfactants, salts, solvents, plasticisers and toxic heavy metals [16,21]. Heavy metals are some of the most problematic environmental pollutants [22]. When the permissible concentration is exceeded, they are toxic to both plants and animals [23]. Heavy metals may also have microbicidal properties [24]. The presence of heavy metals in wastewater is therefore a serious obstacle to the use of biological treatment techniques based on microorganisms sensitive to toxic compounds [25].

Biological wastewater treatment using trickle bed bioreactors is a commonly known and used solution that has a number of advantages, primarily economic ones. Building and maintaining a plant of this type does not involve a large financial outlay compared to other wastewater treatment technologies [26,27]. The biofilm formed on the packing surface plays a key role in biological wastewater treatment systems using trickle bed bioreactors [28]. Diverse species of microorganisms working together during the decomposition of organic substances to maintain vital functions purify the stream entering the bioreactor column [26]. Packing elements of the bioreactor constitute a frame for the formation of a biofilm, which is a natural structure consisting of microorganisms capable of creating aggregated communities [20,29]. Thanks to the layer of biopolymers (EPS) surrounding the cells that form the biofilm, microorganisms are protected against UV radiation, drying, as well as changes in pH, pollutant concentration and temperature [20,30]. The surface of the packaging components must create proper conditions for microorganism colonisation.

The selection of packing elements of the bioreactor is one of the most important factors because packing properties, including specific surface area, affect the efficiency of the process. Increasing the specific surface area of the packing leads to reducing the bioreactor size, which decreases the costs of building the installation [31,32].

The shape and morphology of the packing influence the structure and rate of biofilm formation [20,27,30,33]. The greater the specific surface area of the packing elements in the bioreactor, the greater the area of colonisation by bacteria. A larger adhesion surface of microorganisms results in a larger contact surface of the contaminated stream entering the bioreactor with the microorganisms forming the biofilm [33].

In addition to the parameters of the finished element of the bioreactor packing, the properties of the material from which it is made should be taken into account. Such a material should be low-cost, easily available, insoluble, resistant to various environments, easy to process and non-toxic [34]. Waste is certainly a cheap and widely available material. The possibility of using some waste materials, e.g., used tires [34,35,36,37,38], acrylonitrile butadiene styrene (ABS) from waste electrical and electronic equipment [39], straw [40,41,42] and wood [40,43], as a substrate for biofilm formation has been investigated in the literature. Attempts have also been made to test textile materials, which are not classified as waste materials, as packing elements, e.g., cotton fabric [44] and polyester non-woven fabric [45].

The growth of the world’s population, rising standard of living and so-called “fast fashion” contribute to the constantly increasing consumption of textile materials from various industries [46,47,48,49]. According to estimates by the European Environment Agency (EEA), global fibre production in 2030 will amount to 145 million tons [17]. Consequently, this leads to an increase in the amount of pollutants generated during textile production and an increase in the amount of solid waste in landfills [50,51]. Due to their high impact on the environment, textiles have been identified as a priority group in the development and implementation of new technologies consistent with the principles of the circular economy [50,51]. In the clothing industry, products are mainly made of cotton and polyester [52]. The use of polyester fibres by the textile industry ultimately leads to their release into the environment. The presence of polyester can be observed in both aquatic [18,53] and terrestrial environments [54]. The entry of microtextile waste and microplastics into the aquatic environment is associated with their entry into the food chain [49]. Polyester found in the environment (often in the form of microplastics) comes mainly from the textile industry [55].

Activities related to textile waste management are extremely important in the light of Directive 2018/851 of the European Parliament and Council of 30 May 2018 on waste. From January 2025, EU Member States are to establish a separate collection system for household textiles to ensure an adequate level of recycling.

Mandatory recycling of textile materials opens up a number of possibilities for their use. Textile waste materials are used by the furniture, carpet, paper, textile, automotive and construction industries [56]. There are studies available in the literature on the use of textile waste as fillers to produce composite materials, including partition walls [57], ceiling tiles [58], acoustic panels [59,60], thermal insulating materials [61,62], cement mortars [63] and as an addition to concrete [64,65].

Previous research suggests the possibility of using non-waste materials from the textile industry, including polyester materials, as packing elements in biological wastewater treatment [44,45]. Therefore, in this study, for the first time, the applicability of textile waste material as packing elements of the bioreactor to the treatment of real textile wastewater was investigated. The proposed solution would enable simultaneous management of textile waste and wastewater treatment, which is consistent with the principles of a circular economy. Moreover, the selected waste belongs to the group of cheap and easily available materials, which have the potential to be used on a wider scale.

## 2. Materials and Methods

### 2.1. Materials

Post-production waste of textile and polyester products (PES) intended for window blinds was used as the raw material for the bioreactor packing. Waste of various colours was used: green, brown and gray. Textile waste was pre-cut and then crushed in a mill into particles with a diameter of 3 mm and smaller. The composition percentages of the raw material were tested using a method consistent with the PN-P-04604:1972 standard [66]. Fibres were identified on the basis of three tests: flammability, solubility and microscopic observation. A Motic B1 microscope with a melting point testing setup (Precoptic Co., Warszawa, Poland) was used in the tests.

GreenPoxy 56 epoxy resin and SD 7561 (Sicomin, Chateauneuf les Martigues, France) hardening agent were used as a binder for combining waste (matrix). It is a bio-based resin containing up to 51% carbon of plant and animal origin.

Two types of feed supplied to bioreactors were used in the research: model wastewater prepared in accordance with the PN-C-04550-10:1972 [67] standard and real industrial wastewater from textile industry installations. The actual wastewater came from the Experimental Production—Lukasiewicz Research Network—Lodz Institute of Technology, which is involved in, among other things, the production of specialised non-wovens. All wastewater generated during production processes was transported to a physical–chemical pre-treatment plant (coagulation and flotation). A pre-treated wastewater stream was used for the research.

To accelerate the formation of a biofilm on the packing elements, activated sludge was introduced into the bioreactor column. The activated sludge came from a municipal wastewater treatment plant located in central Poland (51°39′57.4″ N 19°28′20.8″ E). The parameters of the activated sludge used were checked: pH was 7.620, conductivity was 1.080 [mS/cm] and dry residue was 1.68 [g/L].

### 2.2. Procedure for Preparing Packing Elements

**Figure 1:**
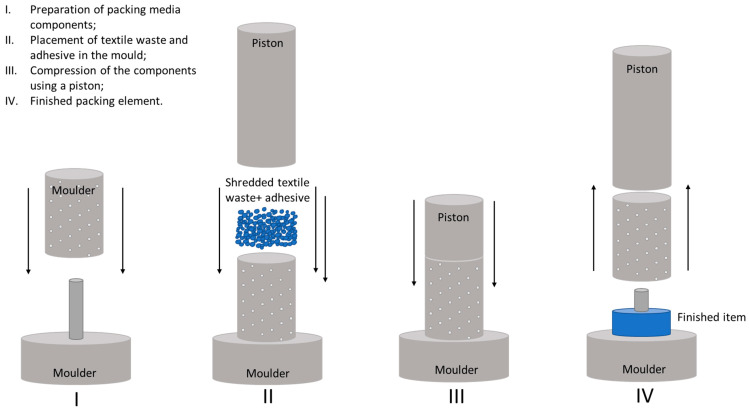
Schematic of the formation of the bioreactor packing elements based on textile waste.

The process of preparing the packing elements for the bioreactor is shown schematically in Figure 1.

A weighed amount of polyester textile waste was mixed with an appropriate amount of epoxy resin. Mixing was carried out in a glass beaker. Then, a small amount of the produced mass was transferred to the mould which shapes the packing. The mould was made of a metal perforated tube 49 mm high and with an 34 mm inner diameter with small apertures on the entire surface. Pressure was provided by a piston with a hole in the head’s centre. The packed bed was formed on a round base with a cylinder of dimensions corresponding to the hole in the piston head with a diameter of 10 mm.

The aim of the process was to form packing elements by pressing and removing any excess resin. The process was carried out at room temperature. After removal from the mould, the elements were conditioned in ambient conditions for at least 48 h, then they were rinsed to remove non-cross-linked compounds. The elements were placed in a tank with distilled water, mixed and left for 24 h and, after that time, the operation was repeated. The elements produced had a diameter of 40 mm and height of 10–15 mm, with a hole in the central part with a diameter of approx. 10 mm. The aim of adding a hole inside the formed element was to increase its specific surface area.

### 2.3. Durability of Packing Elements

The properties of the formed elements and the impact of environmental conditions on their durability were checked.

The element’s ability to absorb water at atmospheric pressure was determined. The samples were weighed, then placed in a beaker with distilled water and weighed again after the determined time. Weight and volume of water absorption were calculated using Formulas (1) and (2) [44,68]:

(1) $$n_{weight}=\frac{m_{w}-m_{d}}{m_{d}}*100\;[\%]$$

(2) $$n_{volume}=\frac{m_{w}-m_{d}}{V}*100\;[\%]$$

where:

*m_w_*—mass of the soaked element;
*m_d_*—weight of the dry element;
*V*—volume of the dry element.

The influence of the aqueous environment on swelling of the element was determined. The samples were placed in a 250 mL cylinder filled with distilled water, and the increase in the element volume was observed after 1, 6, 12 and 40 days.

The hardness of the packing elements was measured using an electronic Shore hardness tester, type C, from Zwick/Roell (Ulm, Germany). In order to determine the impact of the environment on the hardness of the tested elements, both packing elements used in the biological wastewater treatment process and clean ones were examined.

The packing elements were weighed and placed in the bioreactor column. Based on the mass and volume occupied by the packing elements, the bulk density of the bed was calculated according to the following formula:

(3) $$D=\frac{m_{PM}}{V_{PM}}*100\;[\%]$$

where:

*m_PM_*—mass of packing elements placed in the bioreactor;
*V_PM_*—volume of packing elements placed in the bioreactor.

The compressive strength of the packing elements was tested using an INSTRON 3345 camera with Bluehill software (Norwood, MA, USA). Measurements in the axial and radial directions of the tested elements were carried out at a speed of 2 mm/min. The analysis was completed when a load of 5000 N was reached or the sample broke down.

### 2.4. Microbicidal Properties of Packing Elements

The antibacterial effect of packing elements was assessed in accordance with the PN-EN ISO 20645:2006 standard using a representative colony of Gram-negative *Escherichia coli* (*E. coli*)—ATCC 25922 and Gram-positive *Staphylococcus aureus* (*S. aureus*)—ATCC 6538 [69]. The bacterial strains were from Microbiologics (St. Cloud, MN, USA).

The antifungal activity of the formed composites was assessed in accordance with the PN-EN 14119:2005 standard against *Aspergillus niger* (*A. niger*)—ATCC 6275 and *Chaetomium globosum* (*Ch. globosum*)—ATCC 6205 [70]. The fungal strains were from Microbiologics (St. Cloud, MN, USA).

Optical microscopy was used to determine the presence of colonies of *E. coli*, *S. aureus* and fungi *A. niger* and *Ch. globosum*. A Motic 101M microscope (Hong Kong, China) was used for analysis.

### 2.5. Surface Properties of Packing Elements

The contact angle of the tested surfaces was measured with a Linos manual goniometer. The contact angle was determined using the 5-point method using the Surftens Automatik 4.6 program. Each sample was tested at least at 10 points, then the average surface contact angle was calculated based on the results obtained.

The Autosorb-1 apparatus from Quantachrome Instruments (Boynton Beach, FL, USA) was used to determine the specific surface area of the tested elements. The measurements were made using the physical sorption method, and nitrogen was used as an adsorbent at a temperature of 77 K. To determine the specific surface area, the 5-point BET method was used for P/P0 values in the range of 0.10–0.30. The surface was read twice—using a 5-point adsorption isotherm and using a 39-point adsorption–desorption isotherm. Both isotherms were plotted for one sample of the tested material.

The surface morphology of the packing element was analysed using optical microscopy: a Keyence VHX7000N microscope (Osaka, Japan) and a Leica DM6 M optical microscope (Wetzlar, Germany).

The influence of activated sludge and industrial wastewater microorganisms on the chemical structure of the surface of the packing elements was determined. The chemical structure of the samples was assessed by ATR-FTIR spectroscopy in the range of 4000–400 cm^−1^ using a Jasco 4200 spectrometer (Tokyo, Japan) with an ATR Pike Gladi ATR attachment (Cottonwood, AZ, USA).

### 2.6. Assessment of Adsorption of Copper (Cu) Ions by Packing Elements

An element of known weight was placed in a solution with copper content of 5 mg/L. After specified time intervals, samples were taken and the content of copper ions was determined again. Tests for the content of copper ions were performed using Hach Lange cuvette tests: LCK329 and a dedicated Hach Lange DR 3900 spectrophotometer (Düsseldorf, Germany). Based on the loss of Cu ions, the degree of their adsorption on the tested elements was determined.

### 2.7. Characteristics of the Treatment System

The developed biological wastewater treatment technology was based on a bioreactor with a trickle bed bioreactor with vertical flow. Inside the glass column there was a frame made, among others, of PVC mesh, supporting the bioreactor packing which enabled a free flow of liquid.

The stream to be cleaned was fed from the top of the bioreactor using a sprinkler. Due to the gravity force, the liquid flowed through the packed bed, wetting on its way the surface of the packing elements. Ultimately, the stream entered the bottom of the bioreactor column where it was returned to the feed tank. Kamoer peristaltic pumps, model DIP 1500 (Shanghai, China), were used to transport the liquid.

The system was equipped with an aeration system mounted below the packing layer. The aeration system and liquid recirculation operated continuously. The content of the supply tank was replaced cyclically, one cycle lasting from 24 to 72 h.

Three similar bioreactors with different packings were used for the research:

- Reactor 1—elements made of polyester textile waste, 340 pieces weighing 2674.75 g;
- Reactor 2—elements made of polyester textile waste, 340 pieces weighing 2672.05 g;
- Reactor 3—elements of natural origin (LECA^®^), approx. 25 L of lightweight expanded clay aggregates weighing 3695.3 g.

**Figure 2:**
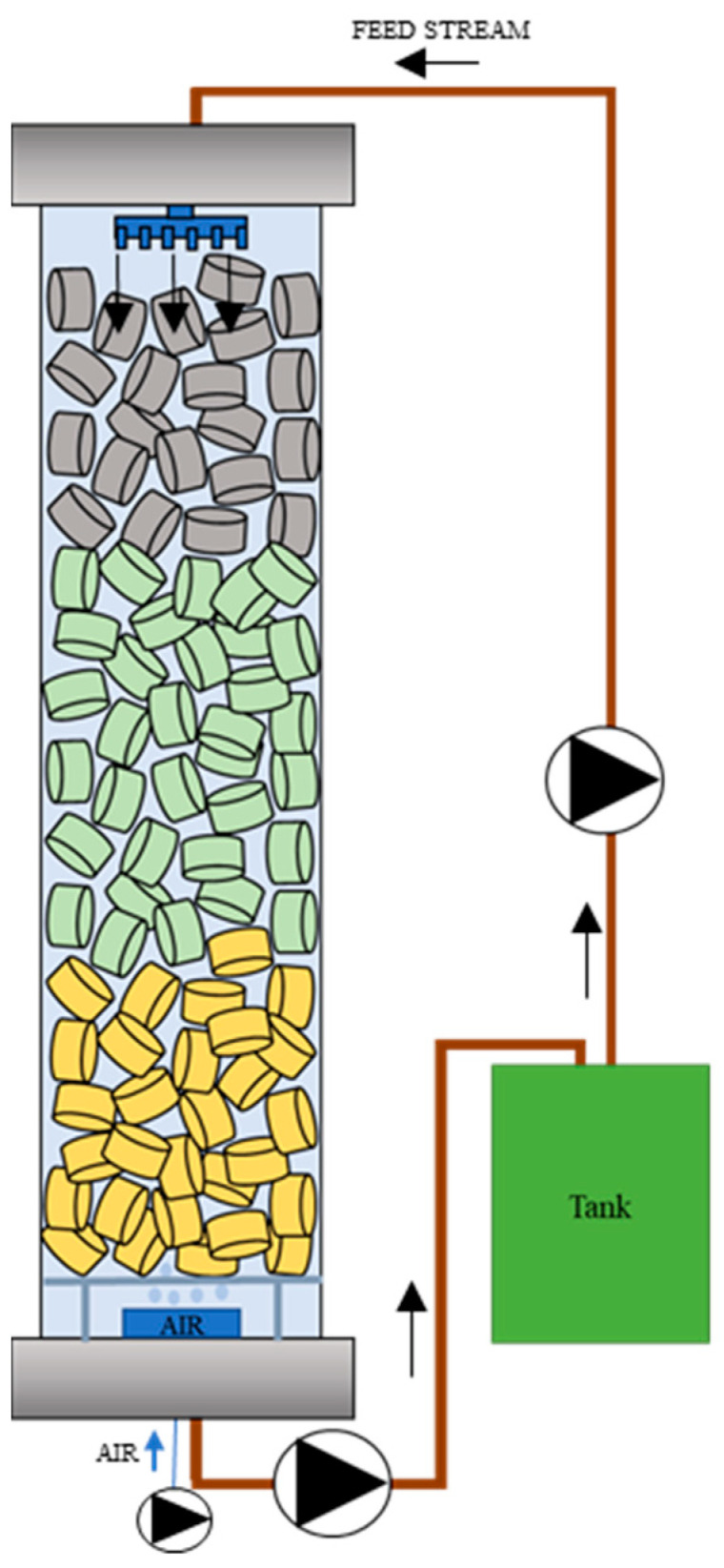
Schematic of the applied purification system with a trickle bed bioreactor.

The treatment system is shown schematically in Figure 2.

The basis of the selected wastewater treatment technology is the formation of a biofilm on the packing elements. In the first stage of the research, model wastewater with a standard weight of reagents in accordance with the PN-C-04550-10:1972 standard was used as the feeding solution [67]. The introduction of a contaminated stream created appropriate conditions for the development of biofilm on the packing elements. Industrial wastewater has a higher load of organic compounds than the model wastewater, therefore the concentration of reagents was increased by 50% over time. Each reactor was fed with a stream of model wastewater for 18 days.

In the next stage of the experiment, in one of the bioreactors containing packing based on textile waste (Reactor 1), the composition of the feed was changed by adding real industrial wastewater. In order to adapt the microorganisms to the new pollutant stream, the percentage of real wastewater in the feed solution was increased at a rate of 10% per week. The feed supplied to Reactor 2 with the packing based on textile waste remained unchanged. The wastewater treatment process using Reactor 3 filled with expanded clay was carried out only in the first stage of the study for comparison purposes.

### 2.8. Methods for the Analysis of Wastewater Composition

The treatment efficiency was assessed based on the change in the chemical oxygen demand (COD) index during one cycle, which was calculated according to the following formula:

(4) $$E=\frac{{COD}_{1}-{COD}_{2}}{{COD}_{1}}*100\;[\%]$$

where:

*COD*_1_—chemical oxygen demand at the beginning of the cycle [mg/LO_2_];
*COD*_2_—chemical oxygen demand at the end of the cycle [mg/LO_2_].

COD tests were performed using Hach Lange cuvette tests: LCK114, LCK314 and a dedicated Hach Lange DR 3900 spectrophotometer (Düsseldorf, Germany).

## 3. Results and Discussion

### 3.1. Characteristics of Materials Used for Packing Elements

Market research shows that textiles are not always produced from homogeneous materials, they are often made of several types of fibres with different properties [71]. Therefore, waste disposed at landfills will differ not only in the types of fibres but also in their content. That is why, before making packing elements, tests were carried out on the materials used in the composite production process. The composition of the textile waste and the surface wettability of substrates used were determined.

**Figure 3:**
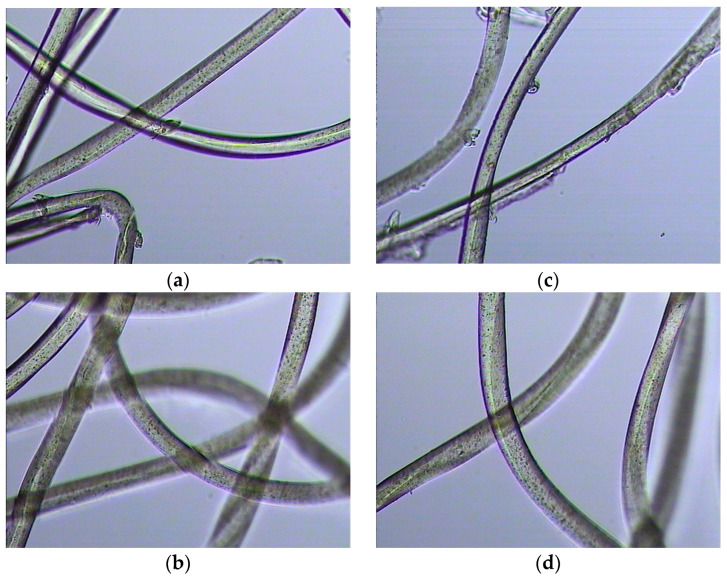
Images of the polyester waste material used to make the packing media. (**a**,**b**) Optical microscope image of the matrix, 200× magnification; (**c**,**d**) Microscopic photo of the thread, 200× magnification.

The waste raw material used to prepare packing elements was identified as 100% polyester (PES). Fibre identification was based on:

- Burning tests: the fibres were burned and melted, forming a slowly cooling mass. After cooling, they formed a ball;
- Solubility tests in chemical reagents: fibres were dissolved in phenol at 75 °C;
- Observation under anoptical microscope: fibres mostly dull with lots of dark spots (Figure 3).

**Figure 4:**
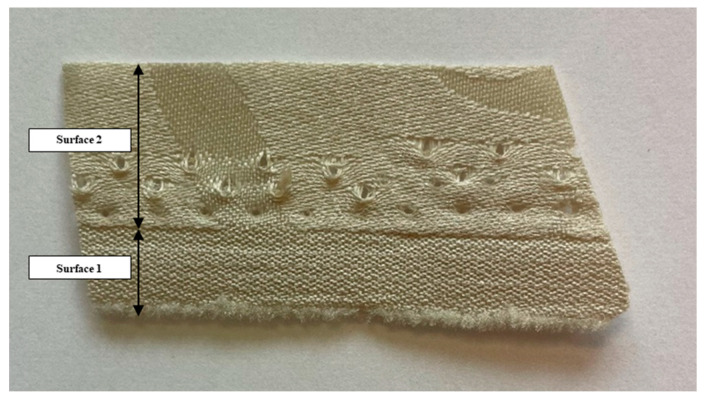
Fragment of waste textile material (green PES). Polyester waste material, before shredding, used to make the packing elements.

The contact angle of the waste textile materials and epoxy resin was tested. The textile waste consisted of two surface fragments with different surface wettability (Figure 4).

The first one was a hydrophilic surface and it was not possible to determine the contact angle. The second fragment was a hydrophobic surface, the values of contact angle are shown in Table 1.

The contact angle of the binder used was checked. After 24 h of contact of the binder surface with the environment, the surface was hydrophilic (average contact angle was 61.26°). In order to check the effect of the binder conditioning time on the surface’s character, the test was repeated after 7 days. The average surface contact angle after a week was 72.67°. The conditioning time had no significant impact on the nature of the surface. Based on the results obtained, the minimum conditioning time for the packing elements was set at 48 h.

### 3.2. Characteristics of Packing Elements—Durability

Packing elements of the bioreactor made of textile waste were subjected to a series of tests to determine their properties and durability in ambient conditions. Before the tests, the durability of the formed elements was analysed. The packing elements were placed in aqueous solutions of different pH: neutral, acidic and alkaline, in the environment of real wastewater and in the presence of microorganisms. The durability of the elements was assessed based on visual, olfactory and tactile sensations. Regardless of the exposure time, the elements did not change their colour and shape, and the binder was not visibly damaged. No noticeable changes in the durability of the packing elements were observed.

**Figure 5:**
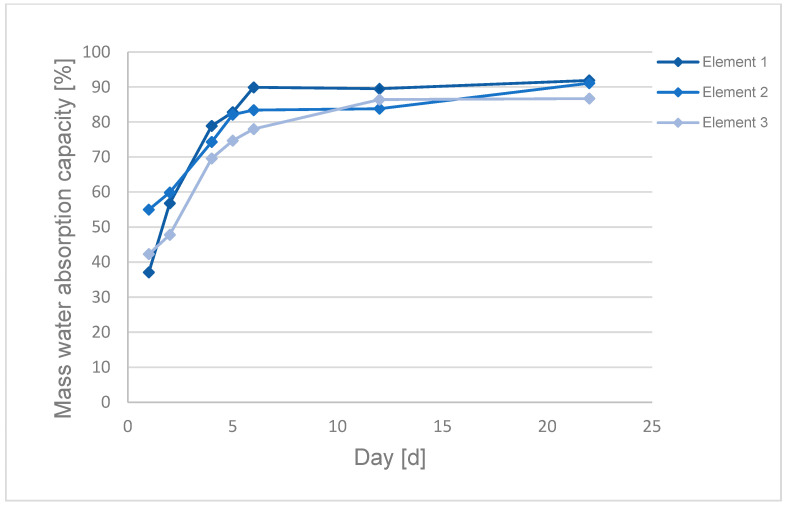
Mass absorption of a packing element based on textile waste.

**Figure 6:**
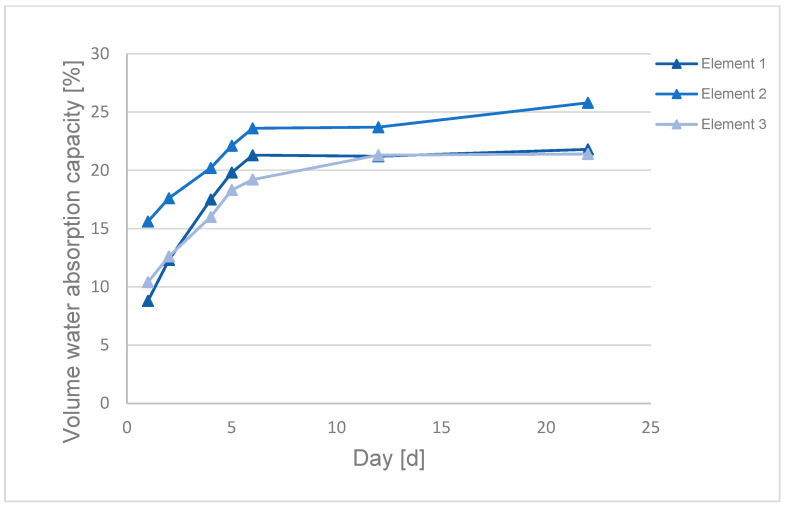
Volume absorption of a packing element based on textile waste.

Water absorption capacity of the packing elements was checked (Figure 5 and Figure 6). The tests were carried out in triplicate as presented in Figure 5 and Figure 6. 

Dry packing elements made from textile waste have low density of approximately 0.2–0.3 g/cm^3^, which results in a significant difference between the obtained values of mass and volume absorption.

Dębska and Lichołai [68] tested water absorption of composite materials made of epoxy resin and plastic waste: polyethylene (PE), polypropylene (PP), polyurethane foam (PU) and expanded polystyrene (EPS). The trend line for absorbability of the composites was similar to the line obtained for the packing elements made of epoxy resin and polyester (PES). Water absorption of the packing elements increased with the duration of their exposure to water. A significant increase in mass and volume water absorption was observed in the first 6 days of the experiment, and on the following days water absorption values did not change significantly.

The higher water adsorption by composites containing PES compared to the composites containing other plastics is caused by the higher content of the plastic used in the composite and its different properties. Differences in mass and volume absorption between the tested packing elements based on textile waste result from differences in their mass, volume and morphology.

High mass absorption values suggest that the elements can almost double their mass in the wastewater treatment process. An increase in the weight of the packing elements increases the weight acting on the elements placed in the lower part of the bioreactor.

Samples saturated with water may change their properties, e.g., reduce their compressive strength or increase their volume, so the influence of water environment on swelling of the packing elements was examined. After 40 days, the elements placed in the cylinders did not swell and did not change their volume. During the wastewater treatment process using trickle bed type bioreactors, the bulk height of the packing elements was checked. This value did not change during the process which confirms the resistance of elements to swelling under the given conditions.

**Figure 7:**
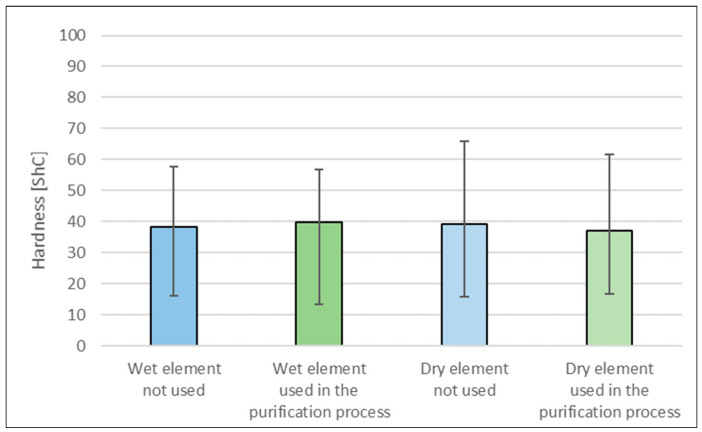
Hardness of packing elements made from textile waste.

Hardness of the packing elements based on textile waste was tested before and after their use in the biological treatment of real textile wastewater (Figure 7).

Average hardness of the elements in all tested variants is comparable. This means that environmental conditions do not change the hardness of the packing element. The elements are resistant to the environment of real wastewater and the presence of microorganisms forming a biofilm on their surface. The large measurement error results from the non-homogeneous surface of the composite.

The compressive strength of packing elements subjected to the rinsing process and used in the biological treatment of textile industrial wastewater was tested in two variants: wet and dry. Because of the random arrangement of packing elements in the bioreactor column, the compressive strength of the elements was tested in both axial and radial directions.

The tested elements showed high compressive strength in the axial direction. Both dry and wet elements involved in the biological wastewater treatment process, as well as clean ones, were characterised by strain strength above >5000 N. In dry samples the displacement was approx. 10–13 mm, while wet samples showed a displacement twice as large, which confirms the influence of water absorption on the mechanical properties of the elements. During the biological wastewater treatment process, the elements were exposed to much lower strains even at an increased scale of the process.

**Figure 8:**
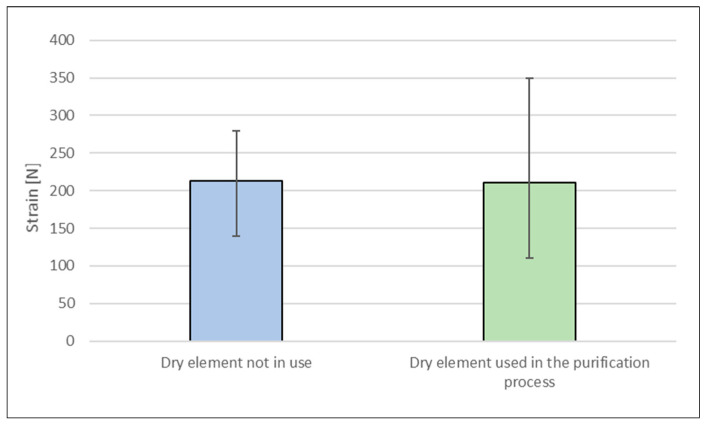
Average strain in the radial direction at which the packing element cracks.

Due to the central location of the hole, the elements tested in the radial direction were characterised by lower compressive strength. The average strain at which dry elements (both new and used in the biological treatment process) cracked was determined. The average strain value for both elements was approximately 200 N (Figure 8).

This means that the process of biological treatment of textile industrial wastewater probably does not deteriorate mechanical properties of the packing elements. Different results obtained by individual packing elements may result from the difference in the height of the tested elements.

**Figure 9:**
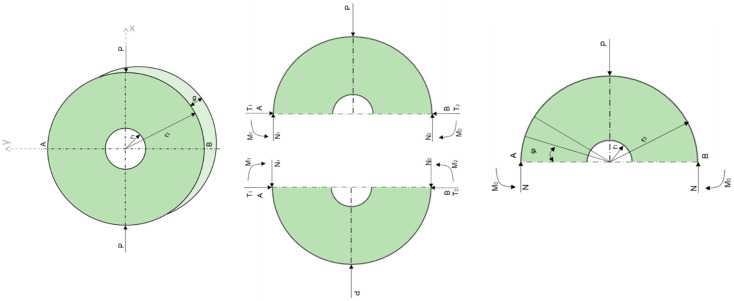
Forces acting on the element during compression in the radial direction [72].

During the process of compressing the composite in the radial direction, several forces acted on the element, as shown in Figure 9.

Durability of the packing element is one of the most important parameters determining its selection [34]. Depending on the type of stream that is purified, packing elements may be exposed to different conditions in which they should demonstrate high durability. Wastewater is supplied continuously to the trickle bed bioreactor, which makes it impossible to replace packing elements during the process. Changing the shape, volume or properties of the packing element may result in deterioration of the efficiency or effectiveness of wastewater treatment during the process.

Moreover, in accordance with the principles of a circular economy, the packing element should have the longest possible life and should be suitable for reuse after the end of the process [73].

The results obtained suggest that the formed elements are characterised by adequate durability and can be used as packing elements in the bioreactor.

### 3.3. Exclusion of Microbicidal Properties of the Element

The microbicidal properties of packing elements were checked in the presence of:

- bacteria: *Staphylococcus aureus* and *Escherichia coli*;
- fungi: *Aspergillus niger* and *Chaetomium globosum*.

The growth of the *S. aureus* strain in the presence of the packing element was checked after 24 h of incubation.

**Figure 10:**
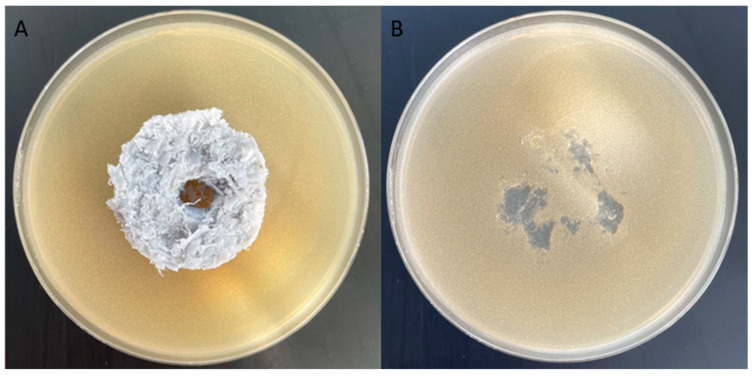
Results of the inhibition zone test on Petri dishes showing no antibacterial effect after 24 h incubation (*S. aureus*). (**A**) Petri dish with a packing element, (**B**) Petri dish after removal of the packing element.

**Figure 11:**
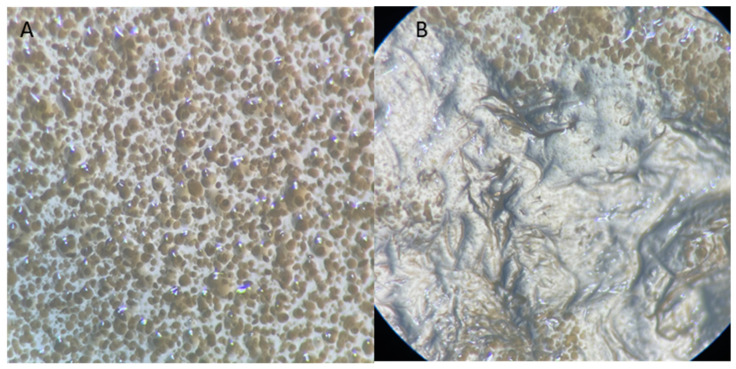
Bacterial biofilm—*S. aureus* after 24 h incubation in the presence of the packing element. (**A**) Substrate next to the element, 50× magnification, (**B**) substrate under the element, 50× magnification.

The surface of the packing element was uneven, therefore locally, in convex places, the pressure of the element on the substrate was greater, which could cause different conditions in given places, e.g., limited oxygen availability. Different conditions could have made it difficult for bacteria to multiply, which explains the lack of uniformity of the bacterial biofilm layer formed on the substrate after removing the packing element (Figure 10B). Formation of the *S. aureus* bacterial biofilm in the vicinity of the tested object suggests that the packing element does not have antibacterial properties. The produced biofilm of *S. aureus* bacteria was observed using optical microscopy. Numerous bacterial colonies were observed around the packing element (Figure 11A) and smaller clusters in places where the uniformity of the bacterial layer was interrupted (Figure 11B). The presence of *S. aureus* bacteria after a 24 h incubation indicates the lack of microbicidal properties of the packing element.

The growth of the *E. coli* strain in the presence of the packing element was checked after a 24 h incubation.

**Figure 12:**
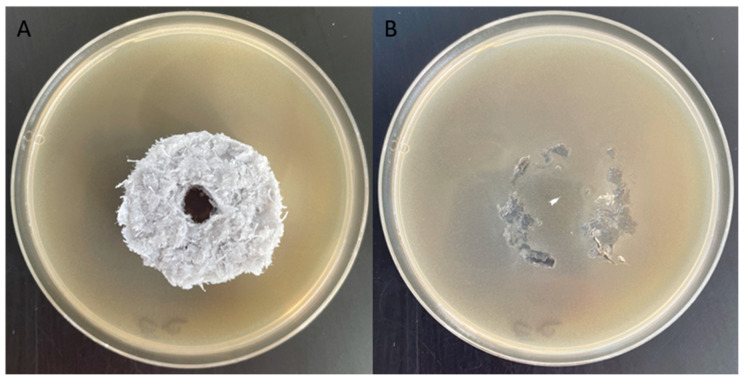
Results of the inhibition zone test on Petri dishes showing no antibacterial effect after 24 h incubation (*E. coli*). (**A**) Petri dish with the packing element, (**B**) Petri dish after removing the packing element.

As in the case of *S. aureus* bacteria, the biofilm of *E. coli* bacteria formed on the substrate is not uniform; the lack of uniformity of the bacterial biofilm produced was visible after the packing element was removed (Figure 12B). The formation of an *E. coli* bacterial biofilm in the vicinity of the tested object suggests that the packing element does not have an antibacterial effect (Figure 12A).

**Figure 13:**
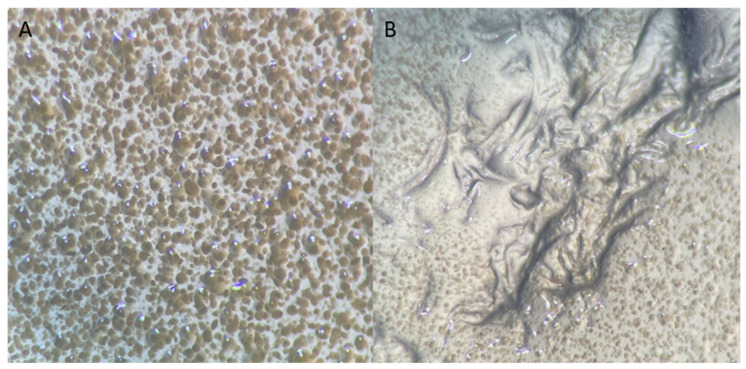
Bacterial biofilm after 24 h incubation in the presence of the packing element. (**A**) Substrate next to the element, 50× magnification; (**B**) substrate under the element, 50× magnification.

The produced biofilm of *E. coli* bacteria was observed using optical microscopy. Numerous bacterial colonies were observed around the mould (Figure 13A) and smaller clusters in places where the uniformity of the bacterial layer was interrupted (Figure 13B). The presence of *E. coli* bacteria after a 24 h incubation proves that microbicidal properties of the packing element are negligible. If the element had microbicidal properties, the bacteria would not occur in its surroundings.

The growth of *A. niger* fungal strains was checked in the presence of the packing element after a 4-day incubation.

**Figure 14:**
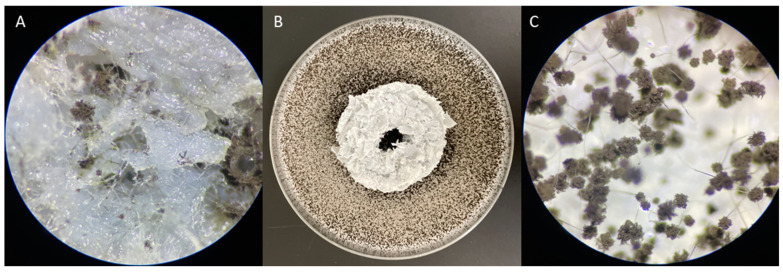
Results of the inhibition zone test on Petri dishes showing no antifungal effect (*A. niger*), (**A**) surface of the element under a microscope, 50× magnification; (**B**) Petri dish with a packing element, 50× magnification; (**C**) area next to the element under a microscope, 50× magnification.

Numerous colonies of *A. niger* fungi were observed around the packing element (Figure 14B,C) and on the surface of the element in contact with the substrate (Figure 14A). The lack of a visible growth inhibition zone of the tested fungal strain and the presence of fungi on the tested sample surface indicate the lack of antifungal properties of the packing element.

The growth of *Ch. globosum* fungal strains in the presence of the packing element was checked after a 14-day incubation.

**Figure 15:**
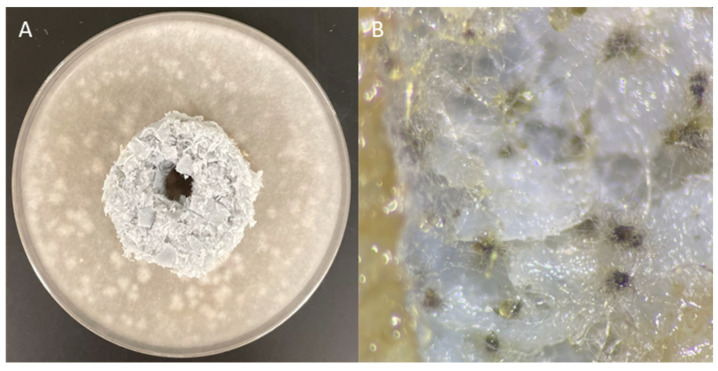
Results of the inhibition zone test on Petri dishes showing no antifungal effect (*Ch. globosum*). (**A**) Petri dish with the packing element, (**B**) surface of the element under a microscope.

Numerous colonies of *Ch. globosum* fungi were observed around the packing element (Figure 15A) and on the surface of the element in contact with the ground (Figure 15B). The lack of a visible growth inhibition zone of the tested fungal strain and the presence of fungi on the tested sample surface suggest that the tested elements do not have microbicidal properties. Due to the height of the element, the lack of moisture and nutrients on its surface and duration of the experiment, both fungal strains were unable to colonise the upper surface of the packing elements.

After analysis of the results, the microbicidal properties of the packing elements were excluded. The results suggest that the elements may have the potential to form a biofilm on their surface and can be used as packing elements for the bioreactor.

The possibility of release of organic pollutants from the elements into the aquatic environment was checked. The results are presented in Table 2.

The value of the COD for elements not subjected to the rinsing process increased after 6 days of the experiment, but on the following days this value did not change significantly. The release of contaminants from the packing elements may have been caused by washing out the remains of uncured binder. The theory is confirmed by the smaller amount of organic compounds released by elements previously subjected to a single rinsing process. Packing elements rinsed twice did not release organic compounds into the environment.

The influence of organic compounds released by the elements on the microorganisms of the activated sludge was checked by placing the elements not subjected to the rinsing process in a flask in the presence of activated sludge microorganisms. After 35 days of the experiment, the degree of purification of the model wastewater was tested over a 24 h period; a degree of pollution reduction above 51% confirmed the presence of microorganisms in the system.

### 3.4. Characteristics of Packing Element Surface

The wastewater treatment technology used is based on the formation of a biofilm on the surface of the packing elements of the bioreactor. The adhesion of microorganisms to the surface of the elements is crucial for the development of the biofilm [74]. The affinity of the surface of elements to the biofilm has an effect on the rate and degree of aggregation of microorganisms [20,75]. Surface wettability is closely related to surface free energy, which influences the adhesion of microorganisms to the substrate [74].

The wettability of the surfaces of the elements at various stages of the treatment process was examined by determining the contact angle. The initial wettability of the material and the influence of the environment on the tested surface parameter of the elements were determined. The new packing element was characterised by a hydrophobic surface with the wetting angle of 102.1–110.3°.

After rinsing (removing non-cross-linked compounds from the element surface), the nature of the surface changed to strongly hydrophilic (it was not possible to place drops). The hydrophilicity of the material promotes the colonisation of microorganisms on the surface of the manufactured elements [75,76]. The surface of elements exposed to the real wastewater environment also showed a hydrophilic character. Similar results were obtained for elements involved in the biological wastewater treatment process. This means that after rinsing, the hydrophilic surface of the elements does not change its character regardless of the environmental conditions.

Another important property of packing elements is the specific surface, which is the sum of the external and internal surfaces of the tested material [77], so its size is influenced by the shape, porosity and surface morphology of the tested object [78]. 

Mohamed S. Hellal et al. used non-woven polyester as a biofilm carrier, assuming that the material itself has a very large surface area, without considering the structure and pore size of the material [45]. In this study the size of the specific surface area of the packing elements based on textile waste was determined, considering the structure and pore size of the material. The addition of resin certainly alters the surface of the polyester itself; however, the shaping of the parts can cause voids to form in the structure. The average specific surface area of the element not subjected to the rinsing process was 0.1595 m^2^/g. This means that the elements are not porous materials. Due to the method of production and the binder used, there was no large amount of free space in the structure of the produced composite. Washing out non-cross-linked compounds from the element during the rinsing process did not change its specific surface. The average specific surface area of the washed element was 0.1521 m^2^/g, which is a similar value within the margin of error.

However, the average specific surface area of the element used in the biological treatment of real wastewater decreased to 0.0788 m^2^/g. The difference in the size of the specific surface of the tested packing elements is caused by the presence of a biofilm in the structure of the composite used in the biological treatment process. The formation of a biofilm on the rough surface of the composite resulted in a reduction of its specific surface area.

Porosity is a parameter that increases the specific surface area of the element and accelerates the maturation process of the biofilter [33]. The total pore volume of the tested element was only 3.4 × 10^−4^ cm^3^/g, where the average pore size was approximately 25.81 nm. The structure of the element promotes attachment and formation of biofilm. However, the small diameters of the channels limit the access of media such as air or the stream of introduced liquid to the interior of the elements, which creates different conditions [79]. Moreover, the inner surface of the pores may become clogged due to their size.

**Figure 16:**
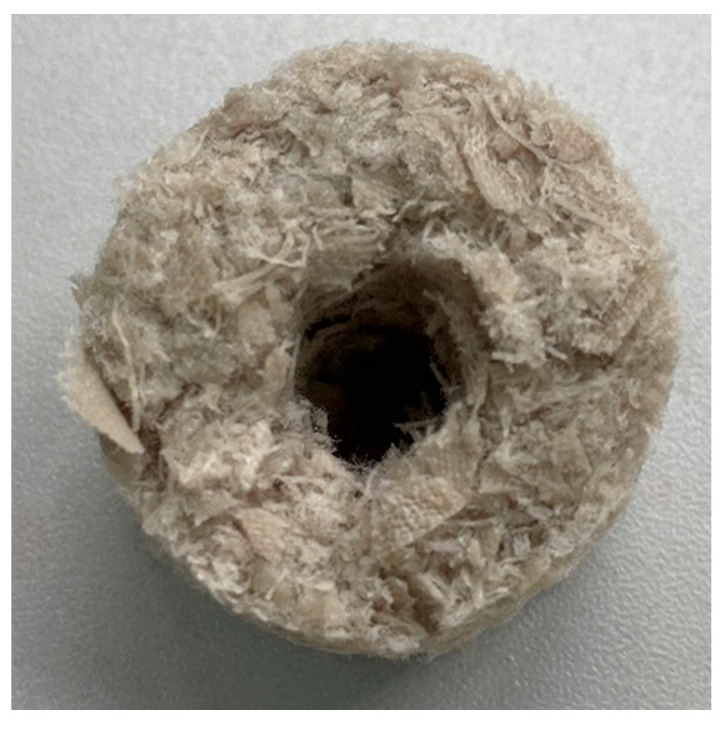
Photo of the packing element (green PES).

The specific surface area of the manufactured elements was increased by placing a hole with a diameter of approx. 10 mm in the central part (Figure 16). In this way, the area of adhesion of microorganisms to the produced packing elements of the bioreactor was increased.

**Figure 17:**
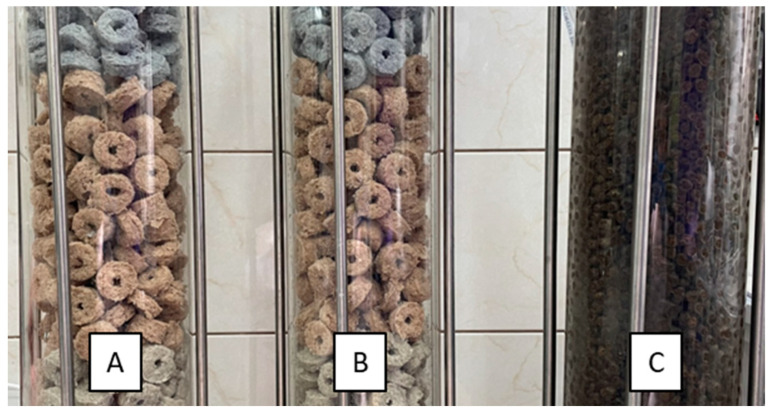
Applied packing elements placed in bioreactor columns. (**A**) Reactor 1, (**B**) Reactor 2, (**C**) Reactor 3.

The shape of the elements made it possible to form empty spaces between the elements placed in the reactor column (Figure 17). The voids facilitated the flow of air and liquid through the column and prevented the biofilter from clogging [31]. The bulk density of the packing elements was calculated to be approximately 487.4 g/dm^3^.

**Figure 18:**
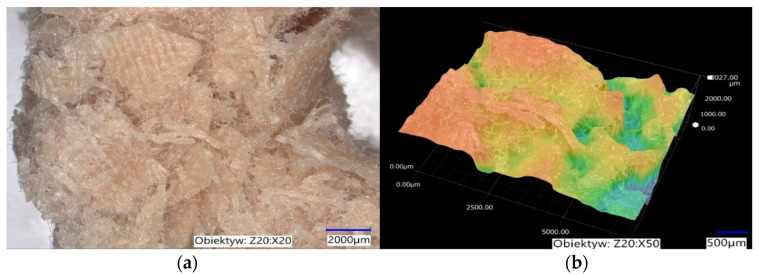
(**a**) Photo of the surface of the packing element at 20× magnification; (**b**) 3D profile of the element surface magnification.

The packing elements produced from textile waste are characterised by negligible porosity, but their surface is not smooth. A 3D model of the packing element was made using the acquired optical microscopy images (Figure 18), which shows the rough nature of the element’s surface. Surface irregularities cause increased adhesion of the biofilm, making it difficult to detach during the process.

The influence of the biological treatment process of real wastewater and the components involved in it on the surface of the elements was examined.

**Figure 19:**
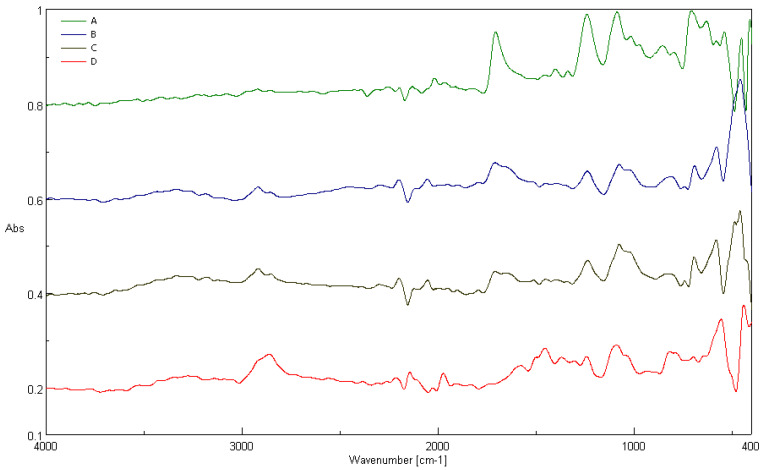
Fourier transform infrared spectroscopy (FTIR) spectra of: (A) untreated polyester fabric and (B,C) packing elements (D) epoxy resin.

The chemical structure of the new element and the element used in the biological treatment process was analysed. Figure 19 shows characteristic FTIR bands ν [cm^−1^] determined for a reference sample of polyester material (sample A), an unused packing element (sample B), an element used in the biological treatment of real wastewater (sample C) and a binder used to produce composites (sample D—resin epoxy GreenPoxy 56). Peaks in the FTIR spectra of the tested samples shown in Figure 19, as A, B, C and D, appeared in the range of 4000–400 cm^−1^.

In spectra A, B and C, four main absorption bands can be observed: ~2900 cm^−1^, ~1700 cm^−1^, ~1200 cm^−1^, ~1100 cm^−1^. These peaks come from O-CH_3_/C-OH, C=O, C-C-O and O-C-C stretching, respectively [80]. FTIR spectra show typical signals for polyester material in the following areas: 1700 cm^−1^—C=O from bond vibrations, 1200 cm^−1^ from the aromatic ring, 1100 cm^−1^ from O=C-O-C, 967 cm^−1^ from C=C stretching and 869 cm^−1^ from hydrogen in benzene [81,82]. The peaks around 2900 cm^−1^ are attributed to the C(sp3)-H stretching of the epoxy resin (SR GreenPoxy 56) as the polymer binder (Figure 19, D) [83,84,85]. Additional small signals in the 2000 cm^−1^ and 2500 cm^−1^ areas appearing for sample C (Figure 19, C) may result from the influence of real wastewater or microorganisms on the tested element. Spectra B and C are characterised by a similar number and size of peaks, which indicates a negligible impact of biological wastewater treatment on the chemical structure of the packings’ surface.

**Figure 20:**
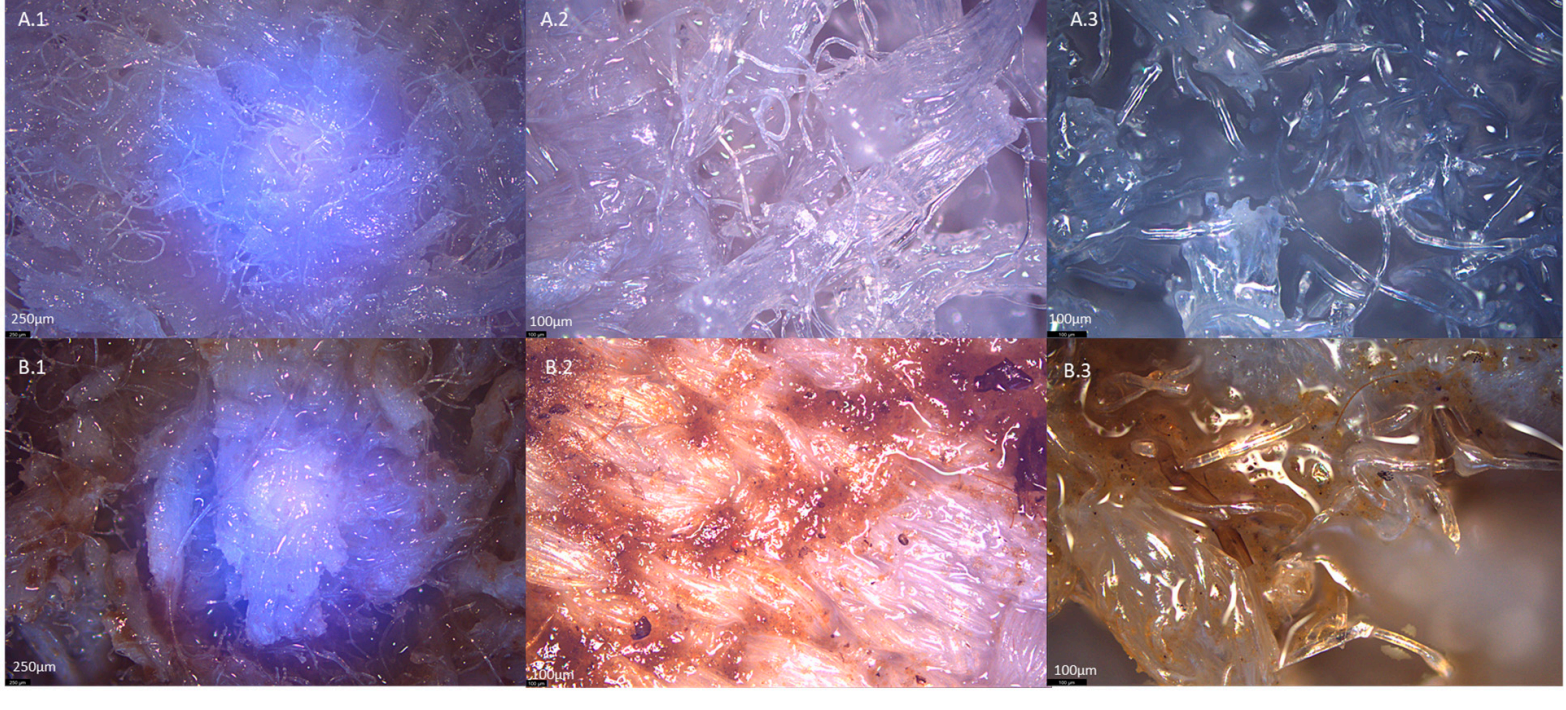
Optical microscope images of the surface of packing elements based on textile waste before (**A.1**–**A.3**) and after (**B.1**–**B.3**) the process of biological treatment of real wastewater. 1—2.5× magnification, 2—5× magnification, 3—10× magnification.

Figure 20 shows images of the surface of the new element and the element used in the biological treatment process at various magnifications.

No significant changes were observed during the optical analysis of the surfaces of the compared elements; in both cases, fragments of polyester waste, single polyester fibres and epoxy resin were visible. The photos of the element used in the biological treatment process (Figure 20(B.1–B.3)) show the remains of the biofilm formed on its surface during the experiment. The presence of the biofilm confirms applicability of the elements.

**Figure 21:**
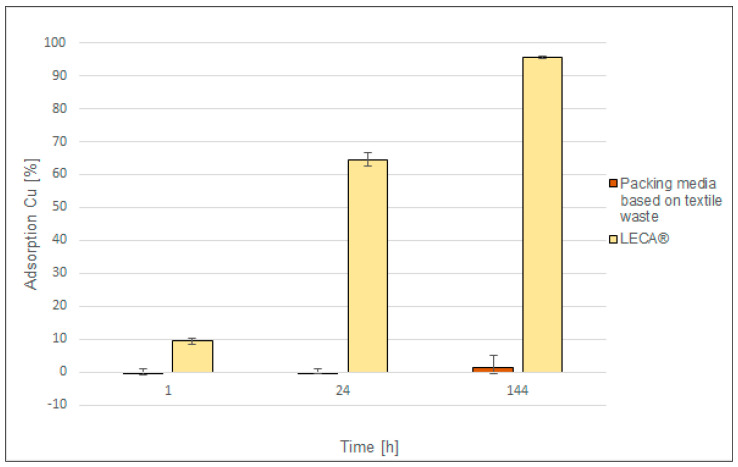
Cu adsorption by different types of packing media: textile waste elements and LECA^®^.

The ability of the elements to adsorb copper was tested. For comparative purposes, analogous tests were carried out for an expanded clay (natural LECA^®^ ceramsite). Then, based on the results obtained (Figure 21), adsorption properties of the tested packings were compared.

Packing elements based on textile waste did not demonstrate copper adsorption capacity. The tests showed that after 6 days these elements adsorbed less than 2% of copper ions from the standard solution, unlike expanded clay LECA^®^ which after 6 days adsorbed over 95% of Cu ions.

The adsorption of copper or other heavy metals by the packing elements may change their properties. An excess of heavy metals, e.g., copper, may affect the microbicidal properties of the manufactured elements. Too high concentrations of heavy metals may prove fatal to the resulting biological film, making biological treatment impossible.

### 3.5. Assessment of the Potential of Packing Elements Based on Textile Waste as a Substrate for Biofilm Formation

The potential of the prepared packing as a biofilm carrier in the bioreactor was assessed and compared with natural packing (LECA^®^).

**Figure 22:**
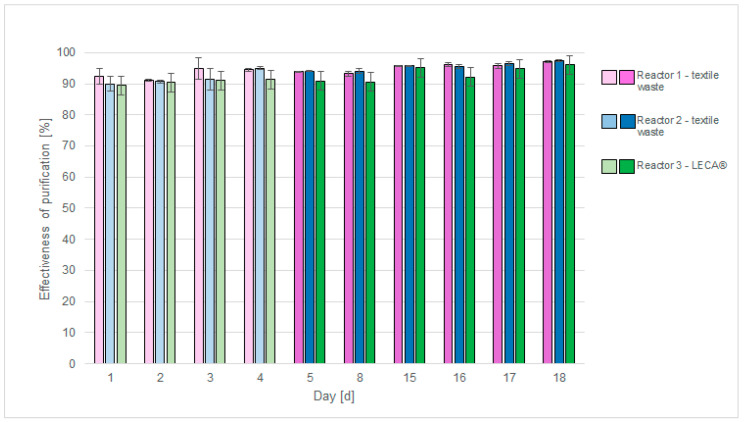
Graph showing treatment efficiency of a model wastewater stream using trickle bed bioreactors with different packings.

In the first stage of the research, aimed at forming a biofilm on the packing elements, high efficiency of removing organic compounds from model wastewater was observed. Taking into account the above and the fact that real textile wastewater is characterised by high COD values, the content of individual components of the model wastewater was increased by 50%, which is marked on the chart with a colour change (from light to dark) (Figure 22).

The biofilm is considered fully formed after 30 days of storage under stable environmental conditions [44]. The aim of this study was to determine the potential for biofilm formation of the fabricated packing media, for which reason the tests were carried out beginning in the first days of microbial aggregate formation on their surface. The results were satisfactory, allowing the potential of the elements as biofilm medium to be determined.

In order to compare the results obtained, tests were carried out using types of packing media: elements based on textile waste materials and LECA^®^.

Literature data reports show that LECA^®^ is a material used in biological wastewater treatment technologies. K. Paździor et al. conducted a biological treatment process for toxic textile wastewater using biological aerobic filters (BAFs) filled with LECA^®^. The authors evaluated its potential as a biofilm medium—the biofilm formed on its surface was characterised by a high diversity of fungi and bacteria. During the process, an efficiency of purification of organic compounds of about 62% was obtained [86].

It has also found its application in activated sludge technologies as a biomass microcarrier to increase process efficiency. A. Masłoń et al. evaluated the effect of adding powdered LECA^®^ to wastewater treatment in a sequencing batch reactor (SBR). They found that LECA^®^ has properties that support biological wastewater treatment processes [87].

Reactors filled with textile waste (Reactors 1 and 2) were characterised by slightly better purification efficiency of the introduced wastewater stream compared to the reactor filled with LECA^®^ (Reactor 3). The efficiency of purification of organic compounds in both cases was above 90%. This means that elements made from textile waste have the potential to fill a trickle bed bioreactor. The literature provided information on the possibility of using textile materials in municipal wastewater treatment. 

Mansuur Husein et al. used modified waste cotton cloth (MWCC) to remove organic compounds from domestic wastewater using a biological contact reactor (BCBR). With longer treatment time (compared to the process carried out in the study)—8 days—the COD removal efficiency was: 88.2% for the unmodified fabric and above 95% for the modified fabric [44]. 

Mohamed S. Hellal et al. used non-woven polyester fabrics (non-waste material) without the addition of a binder in the biological treatment of municipal wastewater, using a passively aerated biological filter (PABF) type reactor. With a shorter HRT time (compared to the process carried out in the study), organic compound treatment efficiencies of 80–90% were achieved [45]. 

The efficiency of purification of organic compounds using trickle bed reactors (Reactors 1, 2 and 3) obtained in this study has similar values to data presented in the literature.

In the following weeks of the experiment, in one of the bioreactors containing packing based on textile waste, the composition of the feed was changed with the addition of real industrial wastewater (Table 3).

Different COD values result mainly from the variability of the composition of real industrial wastewater from textile industry installations. Based on the results obtained during the week, the average treatment efficiency was calculated for the introduced streams differing in the content of real wastewater.

In order to exclude the influence of the environment on the result, it was compared with the effectiveness of purification of a stream which did not contain real wastewater.

**Figure 23:**
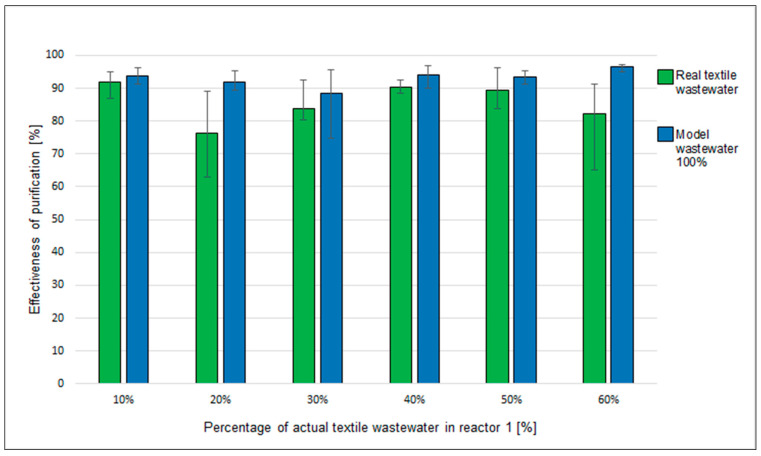
Effectiveness of purification of a real wastewater stream using trickle bed reactors filled with textile waste.

Analysing data in Figure 23, it can be concluded that the process was stable—the average degree of decrease in the index COD from model wastewater exceeded 88%. Despite the introduction of real wastewater with variable composition and small amounts of copper into the reactor, a high degree of COD reduction was achieved (above 75%) throughout the process until reaching 60% real wastewater in the stream flowing to the bioreactor.

This means that the elements have the potential to act as a biofilm carrier and, moreover, they can be used as packing elements for a trickle bed bioreactor for the treatment of industrial wastewater. The proposed technology is an innovative solution (on the use of textile waste materials and type of actual wastewater), not found in the literature, therefore it is not possible to compare the results obtained with those achieved to date. Further research is being conducted on biological treatment of real industrial wastewater using textile-waste-based packing media.

## 4. Conclusions

The purpose of using packing elements was to create a kind of frame for the formation of a biological film on the wetted surface of the moulds. A slimy layer that formed (biofilm) was responsible for purifying the introduced wastewater stream. In this study, the possibility of using textile waste materials to make trickle bed type bioreactor packing media was tested. The use of textile waste materials to make support elements is a new, previously unexplored application. A series of tests on the properties of the composites produced was carried out, which confirmed the possibility of using polyester textile waste to produce packing elements for the bioreactor. 

Investigations of the surface and durability of the formed elements show that they can be used in biological wastewater treatment processes. The elements were resistant to various ambient conditions, had adequate compressive strength, did not have microbicidal properties and did not adsorb pollutants. Their shape and surface morphology suggested that the elements constitute an appropriate substrate for the formation of a biofilm, which is the basis for this type of wastewater treatment technology. 

The use of the elements as a packing of a trickle bed type bioreactor confirmed their potential as a biofilm carrier for both model and real textile wastewater characterised by high variability over time. The treatment efficiency of the model wastewater stream was above 90%, while in the case of the stream containing 60% actual industrial wastewater it was above 80%. The influence of the treatment process and the introduced wastewater streams on the properties of the elements was tested. The chemical structure and nature of the element surfaces did not deteriorate after using the composites in the biological wastewater treatment process, which confirms the possibility of their repeated use.

Currently, humanity is facing many environmental challenges. The world’s dwindling drinking water supply and increasing amounts of waste in landfills are some of the major problems to be faced. The proposed solution for biological wastewater treatment using polyester textile waste is an innovative solution that requires further research and improvement. The use of polyester textile waste is consistent with the principles of a circular economy. The selected waste raw material is inexpensive and easily available, and the use of packing elements based on it will reduce the amount of polyester materials disposed at landfills. Moreover, the use of a bioreactor with the proposed packing may be an element of textile wastewater treatment technology which enables closing of the process water cycle. In the future, it will be possible to replace existing wastewater treatment facilities with the proposed technology, which may involve a reduction in the use of chemicals or a reduction in the consumption of electricity to run the treatment process. The use of trickle bed bioreactors with textile waste packing elements on a larger scale will help reduce the amount of wastewater emitted into the environment and thus minimise phenomena such as land desertification and drinking water deficit, which is extremely important for the environment. The low cost of construction of the plant, its maintenance and the manufacture of the bioreactor may affect the real possibility of implementing the solution on a larger scale.

## Figures and Tables

**Table 1:** Average contact angle of the surface of materials used to produce packing elements in the bioreactor.

Material	PES Green	PES Brown	PES Gray
Average contact angle [°]	105.73	102.08	110.29

**Table 2:** COD measurement results obtained in the experiment.

No.	Type of Element	Type of Solution	COD after 0 Days [mg/L]	COD after 1 Day [mg/L]	COD after 6 Days [mg/L]	COD after 12 Days [mg/L]
1	not subjected to rinsing	distilled water	<15	106 ± 3	121.5 ± 0.5	131.5 ± 2.5
2	rinsed once	distilled water	<15	18.7 ± 0.7	37.55 ± 3.15	69 ± 20.7
3	rinsed twice	distilled water	<15	<15	<15	<15

**Table 3:** Examples of parameters of feed solutions introduced into the bioreactor column during the process.

Parameter	Content of Post-Flotation Wastewater in the Feed Solution [L]	Content of Post-Flotation Wastewater in the Feed Solution [%]	Conductivity [mS/cm]	pH [83,84,85]	Average COD Value [mg/L]
Model wastewater 2	0	0	0.885	7.644	≈400
Industrial wastewater 1	2	10	1.336	9.295	≈380
Industrial wastewater 2	4	20	1.864	9.979	≈349
Industrial wastewater 3	6	30	2.36	10.408	≈384
Industrial wastewater 4	8	40	3.20	10.886	≈490
Industrial wastewater 5	10	50	3.97	11.159	≈534
Industrial wastewater 6	12	60	4.94	11.437	≈458

## Data Availability

Data are contained within the article.
